# Enhanced Formation of Methylglyoxal-Derived Advanced Glycation End Products in *Arabidopsis* Under Ammonium Nutrition

**DOI:** 10.3389/fpls.2018.00667

**Published:** 2018-05-24

**Authors:** Klaudia Borysiuk, Monika Ostaszewska-Bugajska, Marie-Noëlle Vaultier, Marie-Paule Hasenfratz-Sauder, Bożena Szal

**Affiliations:** ^1^Institute of Experimental Plant Biology and Biotechnology, Faculty of Biology, University of Warsaw, Warsaw, Poland; ^2^UMR 1137, INRA, Ecologie et Ecophysiologie Forestières, Université de Lorraine, Nancy, France

**Keywords:** advanced glycation end products, ammonium nutrition, dicarbonyl stress, D-lactate, glyoxalase, methylglyoxal

## Abstract

Nitrate (NO_3_^–^) and ammonium (NH_4_^+^) are prevalent nitrogen (N) sources for plants. Although NH_4_^+^ should be the preferred form of N from the energetic point of view, ammonium nutrition often exhibits adverse effects on plant physiological functions and induces an important growth-limiting stress referred as ammonium syndrome. The effective incorporation of NH_4_^+^ into amino acid structures requires high activity of the mitochondrial tricarboxylic acid cycle and the glycolytic pathway. An unavoidable consequence of glycolytic metabolism is the production of methylglyoxal (MG), which is very toxic and inhibits cell growth in all types of organisms. Here, we aimed to investigate MG metabolism in *Arabidopsis thaliana* plants grown on NH_4_^+^ as a sole N source. We found that changes in activities of glycolytic enzymes enhanced MG production and that markedly elevated MG levels superseded the detoxification capability of the glyoxalase pathway. Consequently, the excessive accumulation of MG was directly involved in the induction of dicarbonyl stress by introducing MG-derived advanced glycation end products (MAGEs) to proteins. The severe damage to proteins was not within the repair capacity of proteolytic enzymes. Collectively, our results suggest the impact of MG (mediated by MAGEs formation in proteins) in the contribution to NH_4_^+^ toxicity symptoms in *Arabidopsis*.

## Introduction

Plants acquire inorganic nitrogen (N) mainly as nitrate (NO_3_^–^) and ammonium (NH_4_^+^). For many plants, the preferred form of N is NO_3_^–^, even though it must be reduced to NH_4_^+^ in energetically expensive reactions before assimilation, whereas the NH_4_^+^ oxidation state eliminates the need for reduction in the cell (Salsac et al., 1987). Excess NH_4_^+^ in the soil leads to severe growth retardation and other toxicity symptoms in many plants commonly referred to as ammonium syndrome (Britto and Kronzucker, 2013). Despite some hypotheses (referred to in Britto and Kronzucker, 2002, 2013; Podgórska and Szal, 2015; Esteban et al., 2016), the underlying mechanisms of NH_4_^+^ toxicity remain unclear.

Although NH_4_^+^ is a toxic compound, it does not accumulate in plant cells to levels that may be dangerous for cell functioning (Britto and Kronzucker, 2002), as it is efficiently incorporated into amino acid structures due to glutamine (Gln) synthetase (GS) activity coupled with glutamine:2-oxoglutarate (2-OG) aminotransferase (GOGAT) activity in the GS-GOGAT cycle. To function effectively, the GS-GOGAT cycle requires the constant availability of 2-OG, which is derived from the mitochondrial tricarboxylic acid cycle (TCA) (Stitt et al., 2002; Foyer et al., 2011). Therefore, an increased demand for carbon skeletons and higher activity of the TCA cycle are expected in response to NH_4_^+^ nutrition. Presumably, the enhanced TCA cycle activity is associated with higher activity of the glycolytic pathway; however, information related to the influence of NH_4_^+^ nutrition on glycolytic activity is still limited. Triose phosphate isomers (TPs), dihydroxyacetone phosphate (DHAP), and glyceraldehyde 3-phosphate (G3P), which are the intermediates of glycolysis and Calvin-Benson cycle, are very unstable molecules, and thus the β-elimination reaction of the phosphoryl group from the common 1,2-enediolate of both trioses may occur, leading to methylglyoxal (MG, CH3COCHO) formation (Martins et al., 2001; Takagi et al., 2014). Therefore, MG occurrence is an unavoidable consequence of glycolytic and photosynthetic metabolism. The production of MG has been estimated to be approximately 0.1–0.4% of the glycolytic flux (Thornalley, 1991), but it varies based on the organism, tissue, cell metabolism, and physiological conditions (Allaman et al., 2015). Under non-stress conditions, the MG concentration in plant tissues remains relatively low but may rise by several fold under stress conditions (Yadav et al., 2005; Hoque et al., 2016). Additionally, the interconversion of DHAP and G3P may be catalyzed by triose phosphate isomerase (TPI, EC 5.3.1.1) present in the cytosol and chloroplasts, which produces MG as a by-product (Phillips and Thornalley, 1993). Phosphate elimination from TPs is considered the major route for MG production under normal physiological conditions (Richard, 1993). However, MG is also produced in different metabolic pathways such as amino acid catabolism and lipid peroxidation (Allaman et al., 2015).

Externally supplied or stress-induced MG causes growth retardation in *Arabidopsis*, rice, tobacco, and tomato in a dose-dependent manner (Yadav et al., 2005; Engqvist et al., 2009; Hoque et al., 2012, 2017; Wienstroer et al., 2012; Kaur et al., 2015b; Welchen et al., 2016). Because of its highly electrophilic nature as an α,β-dicarbonyl ketoaldehyde, MG exhibits cytotoxicity against different macromolecules; for instance, it is able to irreversibly modify amino acid residues in proteins, leading to the formation of MG-derived advanced glycation end products (MAGEs) (Gomes et al., 2006). Modification of arginine and lysine residues by MG principally leads to the formation of N-delta-(5-hydro-5-methyl-4-imidazolon-2-yl (hydroimidazolone 1, MG-H1) and N-epsilon-(carboxyethyl)lysine (CEL), respectively (Li, 2016). Deposition of MAGEs in proteins alters their structure, stability, and function (Silva et al., 2013). Subsequently, MAGEs cause further protein inactivation and oxidative damage in major cell constituents (Hasanuzzaman et al., 2017). Therefore, protein glycation may influence cell metabolism and physiology (Gomes et al., 2007). Cellular destruction is prevented because MG is converted to the less toxic molecules *S*-D-lactoylglutathione (SLG) and D-lactate.

The main MG catabolic pathway in eukaryotic cells is the glutathione (GSH)-dependent glyoxalase (GLXs) system, comprising the enzymes glyoxalase I (GLXI, *S*-D-lactoylglutathione:MG lyase; EC 4.4.1.5) and glyoxalase II (GLXII, *S*-2-hydroxyacylglutathione hydrolase; EC 3.1.2.6) (Thornalley, 1990). In the glyoxalase pathway, GLXI converts the adduct between MG and GSH to SLG, from which D-lactate and GSH are released by GLXII (Thornalley, 1990). Mustafiz et al. (2011) identified eleven genes encoding GLXI-like and five GLXII-like proteins in the *Arabidopsis* genome^1^. More recent studies of Jain et al. (2016) and Schmitz et al. (2017) confirmed GLXI activity of three predicted active GLXI homologs as indicated by phylogenetic analysis (Kaur et al., 2013); the homologs were renamed by Schmitz et al. (2017) as *GLXI.1* (At1g67280), *GLXI.2* (At1g11840), and *GLXI.3* (At1g08110). GLXI.1 localizes to the chloroplast, the GLXI.2 major isoform localizes to the cytosol and its minor isoform to the endoplasmic reticulum, and GLXI.3 is cytosolic or targeted to the chloroplast (Schmitz et al., 2017). Furthermore, from five loci encoding GLXII-like proteins in *Arabidopsis* genome, two were confirmed to not encode functional GLXII: *GLXII.1* (At2g43430), which encodes a protein that exhibits β-lactamase activity (Limphong et al., 2009) and *GLXII.3* (At1g53580) that encodes a protein that acts as a persulfide dioxygenase (Holdorf et al., 2012). The products of *GLXII.2* (At3g10850), *GLXII.4* (At1g06130), and *GLXII.5* (At2g31350) are active GLXII (Norton et al., 1989). GLXII.2 is cytosolic, whereas the GLXII.4 and GLXII.5 splicing forms localize to both the chloroplasts and mitochondria (Schmitz et al., 2017). Biochemical data about the mitochondrial localization of particular GLX isoforms confirmed recent proteomic studies that demonstrated the presence of GLXI.3, GLXII.4, and GLXII.5 isoforms in the *Arabidopsis* mitochondrial complexome (Senkler et al., 2017). An additional glyoxalase enzyme detected in plants, named glyoxalase III (GLXIII or DJ-1), may transform MG directly into D-lactate in a GSH-independent manner, providing a shorter route for MG detoxification (Kwon et al., 2013). Nevertheless, *Escherichia coli* GLXIII (DJ-1/Hsp31/Park7) was recently shown to be a protein deglycase that prevents the accumulation of already formed MG-glycated amino acids by acting on early glycation intermediates and releases unmodified proteins and lactate (Mihoub et al., 2015; Richarme et al., 2015). Therefore, the role of plant GLXIII requires further elucidation.

The formed D-lactate is translocated into the mitochondria for subsequent metabolism. The mitochondrial D-lactate dehydrogenase (D-LDH, EC 1.1.2.4) localized in the intermembrane space catalyzes the oxidation of D-lactate to pyruvate using cytochrome *c* (cyt *c*) as an electron acceptor (Engqvist et al., 2009; Wienstroer et al., 2012; Welchen et al., 2016). Pyruvate, the major product of the MG catabolic pathway, enters into the TCA cycle via acetyl-CoA (Hoque et al., 2016).

In this study, we showed that NH_4_^+^ nutrition leads in *Arabidopsis* to the increased sugar content and enhanced glycolysis that promotes MG production. The upregulation of the glyoxalase pathway in NH_4_^+^-grown plants is insufficient to prevent the accumulation of MG. High MG concentration enhances the formation of MAGEs in proteins. Collectively, the observed changes in MG metabolism might impair plant cell func- tioning, and therefore might contribute to growth retardation.

## Materials and Methods

### Plant Material and Growth Conditions

**FIGURE 1:**
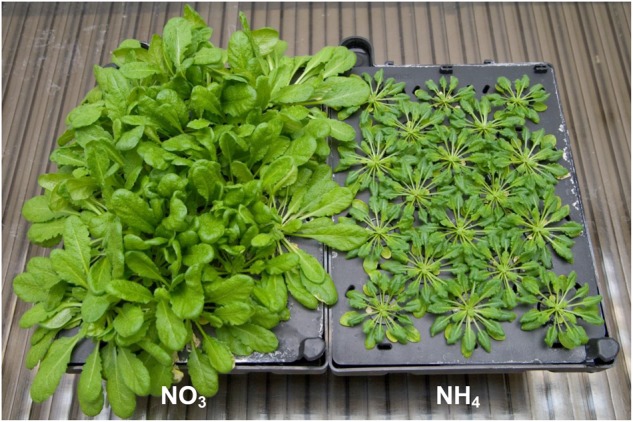
*Arabidopsis thaliana* (ecotype Columbia-0) plants cultured hydroponically for 8 weeks with an 8-h photoperiod on nutrient medium containing 5 mM NO_3_^–^ (NO_3_) or 5 mM NH_4_^+^ (NH_4_) as a sole nitrogen source. Plants grown on NH_4_^+^ showed severe growth retardation as compared to NO_3_^–^-grown plants. Leaf samples for assays were collected in the middle of the light period.

*Arabidopsis thaliana* ecotype Columbia-0 plants were grown hydroponically using an Araponics SA system (Liège, Belgium). Seeds were sown in half-strength Murashige and Skoog (1962) basal salt mixture with 1% agar, and 1 week after germination, deionized water in the hydroponic box was replaced with a nutrient solution. The nutrient composition was: 1.5 mM KH_2_PO_4_; 2.5 mM KCl; 0.7 mM CaSO_4_⋅2H_2_O; 0.8 mM MgSO_4_⋅7H_2_O; 0.06 mM NaFe-EDTA; 5 mM CaCO_3_ (Lasa et al., 2002a) supplemented with a micronutrient mix (0.28 μM CuSO_4_⋅H_2_O, 0.4 μM ZnSO_4_⋅7H_2_O, 0.15 μM KI, 0.20 μM KBr, and 0.20 μM Na_2_MoO_4_⋅2H_2_O) and 2.5 mM Ca(NO_3_)_2_⋅4H_2_O (NO_3_^–^-grown plants) or 2.5 mM (NH_4_)_2_SO_4_ (NH_4_^+^-grown plants) as the N source. Under NO_3_^–^ nutrition plants grow well (**Figure 1**) and are considered the best control plants. Cultivation of *Arabidopsis* in N-free nutrient medium applied in many previous studies as a control is interpreted by our group as a severe stress (Podgórska et al., 2017). In contrast, the use of combined N sources (such as NH_4_NO_3_) would not enable conclusions to be drawn about the influence of the particular inorganic N forms. The nutrient solution was renewed twice a week. Plants were grown for 8 weeks in a growth chamber under an 8-h photoperiod with 150 μmol m^-2^ s^-1^ photosynthetically active radiation (PAR; daylight and warm white, 1:1; LF-40W; Phillips, Pila, Poland), day/night temperature of 21°C/18°C, and approximately 70% relative humidity. Leaf samples from plants for assays were collected in the middle of the light period.

### Determination of Enzyme Activities

ATP-dependent phosphofructokinase (PFK) and PPi-dependent PFK activities were assayed as described by Gibbs et al. (2000). Phosphorylating NAD^+^-dependent glyceraldehyde-3-phosphate dehydrogenase (NAD^+^-GAPDH) and non-phosphorylating NADP^+^-dependent GAPDH (NADP^+^-GAPDH) activities were assayed as described by Bustos and Iglesias (2003). NADP^+^-dependent isocitrate dehydrogenase (NADP^+^-IDH) activity was assayed in isolated mitochondria as described by Rasmusson and Møller (1990). TPI activity was assayed as described by Ito et al. (2003) in tissue extracts prepared according to Dorion et al. (2005) with a minor modification (use of 50 mM triethanolamine buffer; pH 7.4). For measurements of glyoxalase I activity tissue extracts were prepared as described by Chakravarty and Sopory (1998). GLXI activity was assayed in tissue extracts or isolated chloroplasts following the formation of SLG from the adduct of MG and GSH using the glyoxalase I activity assay kit (Sigma-Aldrich, St. Louis, MO, United States). For measurements of glyoxalase II activity tissue extracts were prepared as described by Singla-Pareek et al. (2006). GLXII activity was assayed in tissue extracts or isolated organelles as described by Martins et al. (1999) by the reaction of 5,5′-dithio-bis-2-nitrobenzoic acid (DTNB) with GSH formed from SLG. D-lactate dehydrogenase and mitochondrial respiratory chain complex III activities were assayed in isolated mitochondria as described by Schertl et al. (2014). Decylubiquinone (dUBQ, Sigma-Aldrich) used for complex III activity assay was diluted in 96% (v/v) ethanol and reduced to decylubiquinol as described by Minchenko et al. (2003). Complex IV activity was assayed spectrophotometrically as described by Wigge and Gardeström (1987).

### Measurement of Metabolite Levels

For the extraction of MG and D-lactate, leaf samples were homogenized 1:1 (w/v) in 5% perchloric acid and centrifuged at 13,000 × *g* for 10 min at 4°C. The supernatant was decolorized by charcoal, neutralized by 5 M potassium carbonate solution, and centrifuged at 13,000 × *g* for 10 min at 4°C. The obtained supernatant was used for estimating MG in 100 mM sodium dihydrogen phosphate buffer (pH 7.0), and the reaction was started by the addition of 10 mM *N*-acetyl-L-cysteine. *N*-α-acetyl-*S*-(1-hydroxy-2-oxo-prop-1-yl)cysteine formation was recorded at 288 nm (Wild et al., 2012). At the end of the reaction, 50 nmol of MG was added as an internal standard. MG content was calculated using a known standard curve and expressed as nmol g^-1^ DW. D-lactate determination was conducted spectrophotometrically using bacterial D-lactate dehydrogenase from *Lactobacillus leichmannii* in a coupled reaction involving diaphorase in the presence of NAD^+^ and 3-(4,5-dimethylthiazol-2-yl)-2,5-diphenyltetrazolium bromide (MTT), as described by Monošík et al. (2015). The reaction mixture (1 ml) contained 100 mM phosphate buffer (pH 7.5), 0.4 mM MTT, 0.4 mM NAD^+^, and 0.025 U of diaphorase. The reaction was started by the addition of 0.25 U of D-LDH, and the absorbance was recorded at 565 nm. D-lactate content was evaluated using a standard curve prepared with serial dilutions of stock D-lactate solution to yield a final concentration of 0.5–5 mM. CEL adduct quantification in leaf protein extracts prepared in 50 mM Tris-HCl (pH 7.0) was performed using the OxiSelect CEL ELISA kit (Cell Biolabs, San Diego, CA, United States) with reference to a known CEL-bovine serum albumin (BSA) standard curve. Glucose, fructose, sucrose, and starch contents were assayed as described by Szal et al. (2010) by the methods of Beutler (1985) and Kunst et al. (1985a,b), whereas protein content was assayed as described by Bradford (1976) using BSA as a standard.

### Chloroplast Isolation

Chloroplasts were isolated from 5 g fresh leaves homogenized quickly in a cold mortar with 10 ml of the frozen grinding buffer consisting of 0.33 M sorbitol, 50 mM Hepes buffer (pH 7.3), 0.4 mM KCl, 0.1 mM ethylenediaminetetraacetic acid (EDTA), 0.1% (w/v) BSA, 0.5% (w/v) polyvinylpyrrolidone, and 0.2% (w/v) sodium ascorbate. The homogenate was filtered through 2 layers of polyester vlieseline-type fabric and centrifuged at 500 × g for 5 min. The obtained supernatant was centrifuged again at 3500 × g for 6 min. The resulting pellet was used for the analyses following resuspension in 0.5 mL of buffer containing 0.33 M sorbitol, 50 mM Hepes buffer (pH 7.7), 1 mM EDTA, 1 mM MgCl_2_,1 mM MnCl_2_, and 1% (w/v) BSA.

### Mitochondria Isolation

Intact mitochondria were isolated from rosette leaf tissue as described by Keech et al. (2005) and purified using a discontinuous Percoll density gradient with some modifications (Podgórska et al., 2015). In brief, the gradients were centrifuged at 7,000 × *g* for 40 min, and the mitochondrial fraction that appeared at the interface between the 45 and 30% (v/v) Percoll layers was collected and washed with a solution containing 0.45 M mannitol and 10 mM phosphate buffer (pH 7.5).

### Mitochondrial Oxygen Consumption

Oxygen uptake by isolated mitochondria was measured polarographically using a Clark-type electrode at 25°C (Oxygraph and Oxygraph Plus Software; Hansatech, Norfolk, England) in an incubation medium containing 0.45 M mannitol, 10 mM phosphate buffer (pH 7.2), 5 mM MgCl_2_, 10 mM KCl, and 0.1% (w/v) BSA (Ostaszewska et al., 2014). D-lactate, pyruvate, and *S*-D-lactoylglutathione (5 mM each) were used as substrates, and the oxygen consumption in the presence of 80 μM ADP was recorded. Measurements with inhibitors were performed in the presence of 10 μM antimycin A, 750 μM salicylhydroxamic acid (SHAM), or 5 mM α-cyano-4-hydroxycinnamate (CINN).

### Western Blot Analysis

Protein gel blot analyses were performed using isolated mitochondria or tissue extracts. For cyt *c* level determination, isolated mitochondria (20 μg of protein) were incubated in sodium dodecyl sulfate (SDS)-containing buffer for 40 min at 37°C to weaken protein aggregation and resolved on 16% gel by tricine-SDS-polyacrylamide gel electrophoresis (tricine-SDS-PAGE) as described by Schägger (2006). Purified cyt *c* from bovine heart (1 μg; Fluka Chemical, Milwaukee, WI, United States) served as a protein standard. For PDC determination, 10 μg of mitochondrial protein per lane was separated by SDS-PAGE (10% polyacrylamide), according to a standard protocol. Mitochondrial protein levels were normalized to anti-voltage dependent anion channel 1 (VDAC1). For MG-H1 level determination in tissue extract, 25 μg of protein was loaded on a 14% gel and subjected to SDS-PAGE. The polypeptides were electroblotted on polyvinylidene difluoride membranes using wet transfer (Bio-Rad, Hercules, CA, United States) and probed with anti-cyt *c* (diluted 1:1000; Agrisera, Vännäs, Sweden), anti-VDAC1 (diluted 1:10 000; Agrisera), anti-PDC (kindly provided by Dr. J. Miernyk; Luethy et al., 1995), or anti-MG-H1 (diluted 1:1000; Cell Biolabs) primary antibodies as well as anti-rabbit or anti-mouse secondary antibodies conjugated to horseradish peroxidase (diluted 1:10 000; Bio-Rad). Visualization was performed using the chemiluminescence kit (Clarity Western ECL, Bio-Rad). Signals were detected using the Chemi-Doc imaging system (Bio-Rad). Band density was quantified using QuantityOne 4.6.2 (Bio-Rad). Band density for mitochondrial proteins was determined relative to the value obtained for VDAC1. Total extract proteins (60 μg) along with the molecular marker Precision Plus Protein Standard Kaleidoscope (Bio-Rad) were visualized by colloidal Coomassie blue staining as described by Neuhoff et al. (1990).

### Quantification of Transcript Levels

Total RNA was extracted from 100 mg of leaf tissue using the Syngen Plant RNA Mini kit (Syngen Biotech, Wrocław, Poland). DNase digestion was performed using the RNase-free DNase Set (Qiagen, Hilden, Germany). Complementary DNA was synthesized using the RevertAid H Minus First-strand cDNA synthesis kit (Thermo Fisher Scientific, Waltham, MA, United States). Relative transcript abundance was quantified using comparative quantitation analysis. Transcript content was analyzed using iTaq Universal SYBR Green Supermix (Bio-Rad), according to the manufacturer’s instructions. The primers used in this study are listed in Supplementary Table S1. Genes encoding active GLXs were named according to recently updated nomenclature presented by Schmitz et al. (2017), but previous nomenclature used by Mustafiz et al. (2011) is also indicated. The designed primers spanned exon–exon junctions present in all splice forms of the specific gene. Transcript abundance was quantified by comparing the values obtained for target genes with that of *PP2A* (At1g13320), which served as a reference gene (Czechowski et al., 2005). mRNA quantification and qRT-PCR efficiency of target genes were performed as described by Pfaffl (2001). Transcript levels were expressed in relation to those in NO_3_^–^-grown plants (value of 1).

### Statistical Analysis

Values are the mean ± standard deviation (SD) of three to five independent biological replicates. Protein gel blot/activity analysis was performed using data from at least two independent experiments with a minimum of three biological replicates. Tissue extracts or mitochondria, constituting one biological replicate, were prepared from a single culture. Experimental data were analyzed using Microsoft Excel 2010 (Microsoft Corporation, Redmond, WA, United States). Significant differences were identified using Student’s *t*-test at *P* ≤ 0.05 unless indicated otherwise.

## Results

*Arabidopsis thaliana* grown under long-term ammonium nutrition exhibit severe growth inhibition (**Figure 1**) similarly to our previous observations (Podgórska et al., 2013) and as it was reported for various plant species such as tobacco and cucumber (Walch-Liu et al., 2000; Roosta and Schjoerring, 2007; Hachiya et al., 2012). We suppose that it is an effect of MG accumulation.

### MG Formation in Glycolysis in Response to NH_4_^+^ Nutrition

Because MG production is stimulated by excess carbon flow in the early stages of glycolysis due to enhanced sugar metabolism (Li, 2016), we examined the concentration of carbohydrates and enzyme activity in the glycolytic pathway of NH_4_^+^-grown and NO_3_^–^-grown *A. thaliana* plants.

**FIGURE 2:**
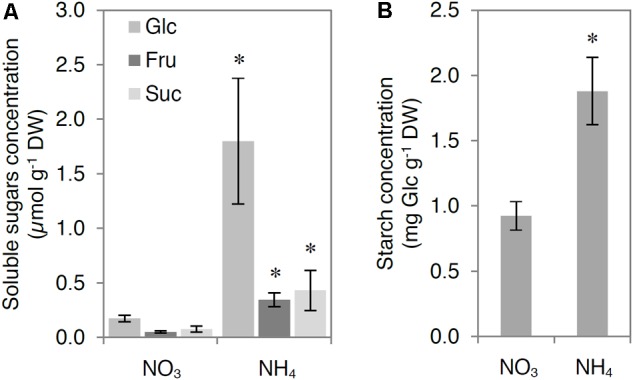
Influence of ammonium (NH_4_^+^) nutrition on carbohydrate levels. Concentration of **(A)** glucose (Glc), fructose (Fru), sucrose (Suc), and **(B)** starch. Leaf samples of nitrate (NO_3_^–^)-grown and NH_4_^+^-grown plants were collected after 4 h of illumination. Values are the mean ± standard deviation (SD) of 4 biological and 2 technical replicates. Significant differences (*P* ≤ 0.05) between NO_3_^–^-grown and NH_4_^+^-grown plants are indicated by an asterisk (^∗^).

Ammonium nutrition increased the concentration of carbohydrates in the leaf tissue. The concentration of glucose, fructose, and sucrose was approximately 10-, 7-, and 6-fold higher, respectively, in the leaf tissue of NH_4_^+^-grown plants compared with that in the leaf tissue of NO_3_^–^-grown plants (**Figure 2A**), whereas the concentration of starch was twofold higher in the leaf tissue of NH_4_^+^-grown plants compared with that in the leaf tissues of NO_3_^–^-grown plants (**Figure 2B**).

**FIGURE 3:**
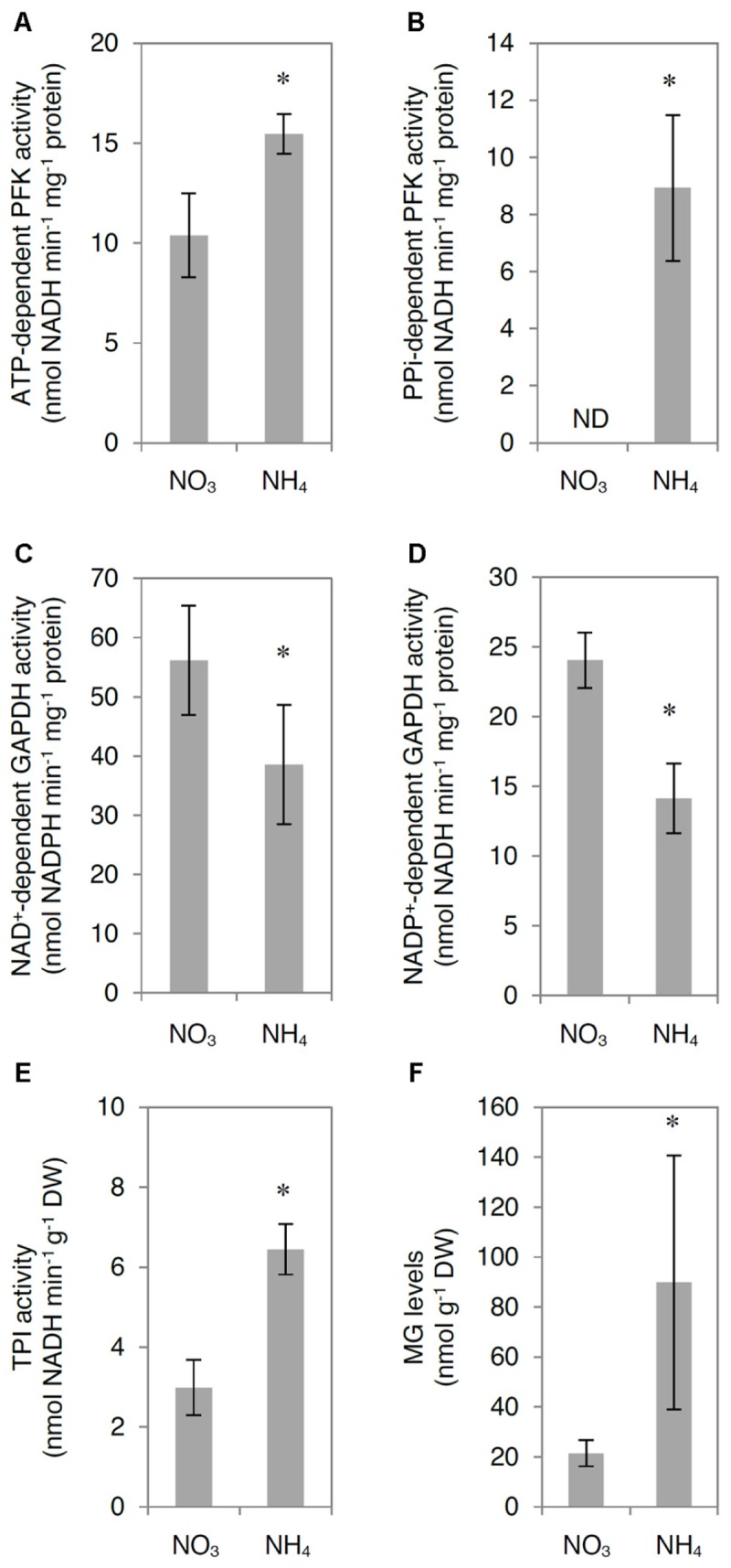
Influence of ammonium (NH_4_^+^) nutrition on glycolytic enzyme activity and methylglyoxal (MG) levels. Activity of **(A)** ATP-dependent phosphofructokinase (ATP-dependent PFK), **(B)** PPi-dependent phosphofructokinase (PPi-dependent PFK), **(C)** phosphorylating NAD^+^-dependent glyceraldehyde-3-phosphate dehydrogenase (NAD^+^-dependent GAPDH), **(D)** non-phosphorylating NADP^+^-dependent glyceraldehyde-3-phosphate dehydrogenase (NAD^+^-dependent GAPDH), and **(E)** triosephosphate isomerase (TPI); and **(F)** MG levels in leaf tissue of nitrate (NO_3_^–^)-grown and NH_4_^+^-grown plants. ND, not detectable. Values are the mean ± standard deviation (SD) of 3–4 biological and 2 technical replicates. Significant differences (*P* ≤ 0.05) between NO_3_^–^-grown and NH_4_^+^-grown plants are indicated by an asterisk (^∗^).

Ammonium nutrition led to modifications in the total activity of glycolytic enzymes in the leaf tissue. The activity of ATP-dependent PFK was higher by approximately 50% in NH_4_^+^-grown plants compared with that in NO_3_^–^-grown plants (**Figure 3A**). Additionally, the activity of PPi-dependent PFK was undetectable in the leaf extracts of NO_3_^–^-grown plants, but present in those of NH_4_^+^-grown plants (**Figure 3B**). However, the activities of phosphorylating NAD^+^-GAPDH and non-phosphorylating NADP^+^-GAPDH were lower by 30 and 40%, respectively, in NH_4_^+^-grown plants compared with those in NO_3_^–^-grown plants (**Figures 3C,D**). The differences in enzyme activities between the early and late stages of the glycolytic pathway might lead to a higher TPs levels, which in turn increase MG generation. The activity of TPI, which catalyzes the equilibrium reaction between G3P and DHAP, was twofold higher in the leaf extracts of NH_4_^+^-grown plants compared with that in leaf extracts of NO_3_^–^-grown plants (**Figure 3E**), confirming the possible enhancement of MG generation. Indeed, MG levels in the leaf tissue of NH_4_^+^-grown plants were more than 4 times higher than those in the leaf tissue of NO_3_^–^-grown plants (**Figure 3F**). The activities of all analyzed enzymes in the TCA cycle were higher in response to NH_4_^+^ nutrition (Supplementary Figure S1).

### MG Degradation Under NH_4_^+^ Nutrition

**FIGURE 4:**
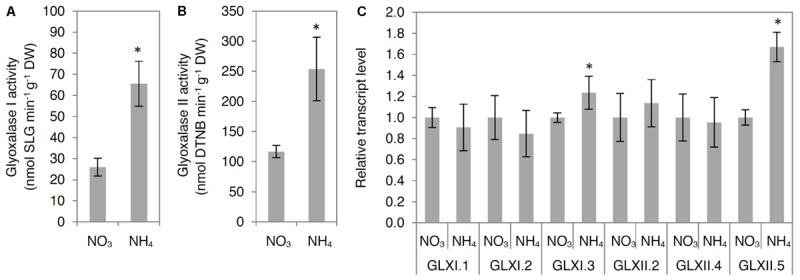
Influence of ammonium (NH_4_^+^) nutrition on principal methylglyoxal (MG) detoxification pathway. Activities of **(A)** glyoxalase I and **(B)** glyoxalase II; **(C)** expression of glyoxalase I and II genes in leaf tissue of nitrate (NO_3_^–^)-grown and NH_4_^+^-grown plants. Values are the mean ± standard deviation (SD) of 3–4 biological and 2 technical replicates. Significant differences (*P* ≤ 0.05) between NO_3_^–^-grown and NH_4_^+^-grown plants are indicated by an asterisk (^∗^). DTNB, 5,5′-dithio-bis-2-nitrobenzoic acid.

To analyze MG degradation in NH_4_^+^-grown plants, we measured in tissue extracts the activities of glyoxalase I and glyoxalase II, constituting the glyoxalase pathway, and determined the transcript levels for all genes encoding for functionally active GLX. The examined GLXI and GLXII activities were enhanced by more than twofold in NH_4_^+^-grown plants compared with those in NO_3_^–^-grown plants (**Figures 4A,B**). Higher activities of GLXI and GLXII were associated with up-regulation of *GLXI.3* and *GLXII.5* (**Figure 4C**). However, the expression of *GLXI.1, GLXI.2*, and *GLXII.4* showed no change in response to NH_4_^+^ nutrition (**Figure 4C**).

**FIGURE 5:**
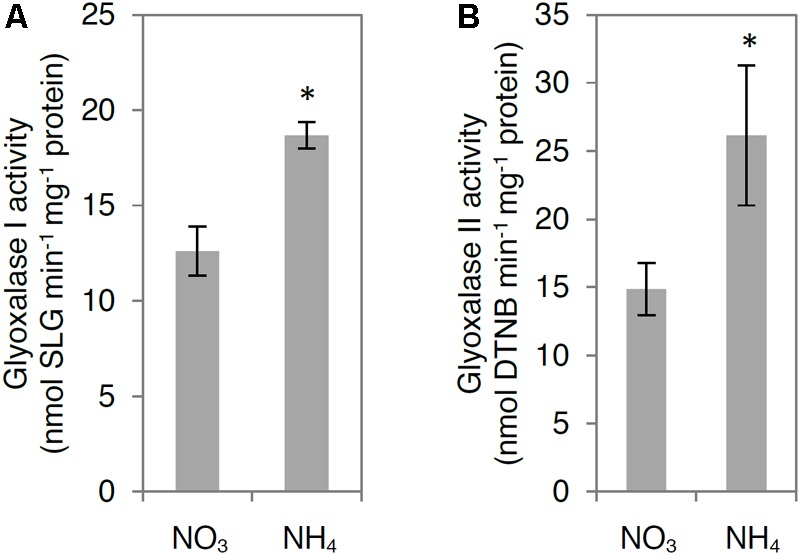
Influence of ammonium (NH_4_^+^) nutrition on chloroplast methylglyoxal (MG) detoxification pathway. Activities of **(A)** glyoxalase I and **(B)** glyoxalase II in chloroplasts isolated from leaf tissue of nitrate (NO_3_^–^)-grown and NH_4_^+^-grown plants. Values are the mean ± standard deviation (SD) of 3 biological and 2 technical replicates. Significant differences (*P* ≤ 0.05) between NO_3_^–^-grown and NH_4_^+^-grown plants are indicated by an asterisk (^∗^).

**FIGURE 6:**
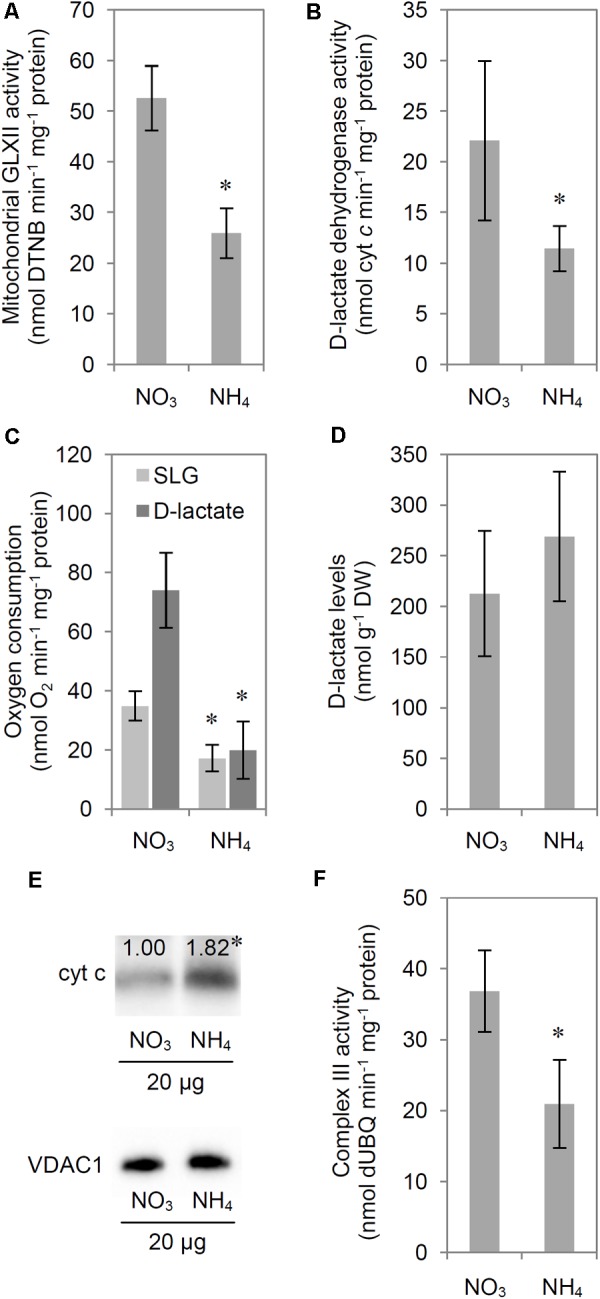
Influence of ammonium (NH_4_^+^) nutrition on *S*-D-lactoylglutathione (SLG) and D-lactate catabolism. Activity of **(A)** mitochondrial glyoxalase (GLX) II and **(B)** D-lactate dehydrogenase; **(C)** mitochondrial oxygen consumption with SLG or D-lactate as substrates; **(D)** D-lactate levels in leaf tissue; **(E)** cytochrome *c* protein levels in isolated and purified mitochondria and **(F)** activity of complex III of the electron transport chain in leaf tissue of nitrate (NO_3_^–^)-grown and NH_4_^+^-grown plants. Proteins from the mitochondrial fraction were resolved by tricine-sodium dodecyl sulfate-polyacrylamide gel electrophoresis, electroblotted on a polyvinylidene difluoride membrane, probed with a polyclonal antibody against cyt *c*, and visualized by chemiluminescence. Signal intensity, corresponding to cyt *c* (molecular mass, approximately 14 kDa) versus mitochondrial porin VDAC1 which was used for protein level normalization, was estimated using Quantity One 4.6.2 after background correction. Results are expressed relative to the control; the amount of protein in mitochondria isolated from the leaf tissue of NO_3_^–^-grown plants was set at 1.00. Representative results are shown. Values are the mean ± standard deviation (SD) of 3–5 biological and 1–3 technical replicates. Significant differences (*P* ≤ 0.05) between NO_3_^–^-grown and NH_4_^+^-grown plants are indicated by an asterisk (^∗^).

Because some GLX family members are present in chloroplasts and mitochondria, we also analyzed GLX enzymatic activities in organelle preparations. Chloroplastic glyoxalase I (chlGLXI) and glyoxalase II (chlGLXII) activities were induced under ammonium nutrition by approximately 50 and 75%, respectively (**Figures 5A,B**). In contrast, mitochondrial GLXII (mtGLXII) was found to be 50% lower in response to NH_4_^+^ nutrition (**Figure 6A**).

### Mitochondrial Metabolism of D-Lactate

The activity of mitochondrial D-LDH, which catalyzes the oxidation of toxic D-lactate, decreased by approximately 50% in response to NH_4_^+^ nutrition (**Figure 6B**). However, measurements of enzyme activities *in vitro* allow to determine only the maximal activity of enzyme in optimal conditions. Mitochondrial metabolism of D-lactate and SLG *in vivo* is strictly connected to mitochondrial respiratory chain functioning, as the electrons are transferred by D-lactate dehydrogenase to the respiratory chain through cyt *c*, therefore we measured additionally mitochondrial oxygen consumption in the presence of externally added D-lactate. Oxygen consumption in isolated mitochondria in the presence of D-lactate was diminished by 75% in NH_4_^+^-grown plants compared with that in NO_3_^–^-grown plants (**Figure 6C**). To ensure that the observed oxygen consumption was the effect of electron transfer from D-lactate to cyt *c* and subsequently to complex IV of the respiratory chain, we performed a control measurement in the presence of 10 μM antimycin A (inhibitor of complex III) and 750 μM SHAM (inhibitor of alternative oxidase). Oxygen consumption with D-lactate after the addition of antimycin A and SHAM was the same as that observed in the presence of D-lactate alone, confirming that the measured oxygen consumption was related to D-lactate oxidation (Supplementary Figure S2). Considering that the product of D-lactate oxidation by D-LDH is pyruvate, we also measured the mitochondrial oxygen consumption using pyruvate as a respiratory substrate. Pyruvate oxidation was similar in NH_4_^+^-grown and NO_3_^–^-grown plants (Supplementary Figure S2). Lower D-lactate oxidation could directly depend on metabolic adaptation to long-term growth on a specific N source or it could be a result of changes in D-lactate transport into the mitochondria. Therefore we performed an additional measurement of oxygen consumption with SLG as a substrate. Measuring mitochondrial oxygen consumption in the presence of externally added SLG we were imitating the *in vivo* conditions when SLG is translocated into mitochondria and has to be first converted by mtGLXII to D-lactate and then by D-LDH to pyruvate. The resulting SLG oxidation by isolated mitochondria was 50% lower in NH_4_^+^-grown plants compared with that in NO_3_^–^-grown plants (**Figure 6C**). Externally added α-cyano-4-hydroxycinnamate (CINN; 5 mM), which inhibits D-lactate uptake by the rat brain and heart mitochondria (Ling et al., 2012), failed to inhibit oxygen consumption in the presence of D-lactate in *A. thaliana* (Supplementary Figure S3).

D-lactate concentration in leaf tissue extracts was similar in NH_4_^+^-grown and NO_3_^–^-grown plants (**Figure 6D**). Concurrently, the cyt *c* protein levels increased by 80% in response to NH_4_^+^ nutrition (**Figure 6E**). However, the oxidation of toxic D-lactate requires the oxidized form of cyt *c*. The redox state of the cyt *c* pool depends on the balance between complex III and complex IV activity. Because direct estimation of the cyt *c* pool redox state is impossible, we measured the capacity of complex III and complex IV in mitochondria isolated from NH_4_^+^-grown and NO_3_^–^-grown plants. Our results indicate that complex III capacity decreased by 43% in response to NH_4_^+^ nutrition (**Figure 6F**). Complex IV activity remained stable (NH_4_^+^-grown plants, 1.018 ± 192 nmol cyt *c* min^-1^ mg^-1^ protein; NO_3_^–^-grown plants, 1.035 ± 388 nmol cyt *c* min^-1^ mg^-1^ protein), and these results were in agreement with those reported previously by our group (Podgórska et al., 2013).

### Formation of MAGEs and Protease Activity

Because increased production of MG may affect the formation and degradation of MAGEs, we analyzed the MG-derived modifications of arginine and lysine residues (MG*-*H1 and CEL levels, respectively) and the total activity of proteolytic enzymes in leaf tissue extracts.

**FIGURE 7:**
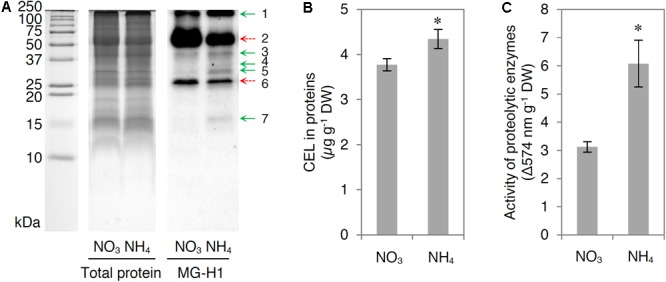
Influence of ammonium (NH_4_^+^) nutrition on methylglyoxal (MG)-derived advanced glycation end products formation and proteolysis. **(A)** Total protein stained gel and MG-derived hydroimidazolone 1 (MG-H1) levels in proteins; **(B)** concentration of N-epsilon-(carboxyethyl)lysine (CEL) in proteins; and **(C)** activity of proteolytic enzymes in leaf tissue of nitrate (NO_3_^–^)-grown and NH_4_^+^-grown plants. Total leaf extract proteins (25 μg for blotting or 60 μg for protein staining) were separated by sodium dodecyl sulfate-polyacrylamide gel electrophoresis. For MG-H1 detection, proteins were electroblotted on a polyvinylidene difluoride membrane and probed with a monoclonal antibody. Representative results are shown. The difference in the levels of MG-H1 between NH_4_^+^-grown and NO_3_^–^-grown plants is indicated by arrows; increased levels are marked with green arrows, whereas decreased levels are marked with dashed red arrows. Values are the mean ± standard deviation (SD) of 3 biological and 1–3 technical replicates. Significant differences (*P* ≤ 0.05) between NO_3_^–^-grown and NH_4_^+^-grown plants are indicated by an asterisk (^∗^).

Distinct levels of MG-H1-modified proteins were detected in response to NH_4_^+^ nutrition (**Figure 7A**). The majority of detected bands (molecular mass higher than 100 kDa, 25–50 kDa, and an additional band of approximately 15 kDa) consistently showed accumulation of MG-H1 residues (**Figure 7A** and Supplementary Table S2), whereas an abundant protein band with a molecular mass of approximately 25 kDa showed decreased MG-H1 levels (**Figure 7A**). Additionally, the levels of the most pronounced band (molecular mass of approximately 50 kDa) with MG-H1 modifications were reduced in response to NH_4_^+^ nutrition, which at least partially resulted from the amount of this particular protein as revealed the total protein staining (**Figure 7A**). Cellular CEL levels were higher by 15% in NH_4_^+^-grown plants compared with those in NO_3_^–^-grown plants (**Figure 7B**). Consequently, NH_4_^+^ nutrition resulted in a twofold increase in the activity of cellular proteolytic enzymes (**Figure 7C**).

## Discussion

### Disequilibrium in Carbohydrate Metabolism Leads to MG Overproduction

The MG concentration in NH_4_^+^-grown plants was markedly higher than that in NO_3_^–^-grown plants (**Figure 3F**), despite the strongly enhanced detoxification by the glyoxalase pathway (**Figures 4, 5**). The production of endogenous MG originates mainly from carbohydrate catabolism. Carbohydrate levels were higher in NH_4_^+^-grown plants than in NO_3_^–^-grown plants (**Figure 2**). Similarly, Walch-Liu et al. (2000) reported that NH_4_^+^ nutrition through the roots increased the levels of hexoses and starch in the aerial parts of the plant. TPs accumulation and subsequent MG production can occur by increased breakdown of carbohydrates in the glycolytic pathway and the reduced ability of glycolytic enzymes downstream of TPs to tackle the increased flux. Therefore, activity of GAPDH is an important regulator of intracellular MG content, as it partially controls the levels of DHAP, a primary source of MG (Kaur et al., 2016). In our study, NAD(P)^+^-GAPDH activity was lower (**Figures 3C,D**), whereas ATP-dependent PFK and PPi-dependent PFK activities were enhanced (**Figures 3A,B**), probably leading to higher levels of TPs and consequently higher activity of cytosolic TPI. Indeed, TPI activity was increased in NH_4_^+^-grown plants (**Figure 3E**). The enhanced generation of MG led to increased MG concentration in the leaf tissue (**Figure 3F**). Previous studies showed that a linear concentration-dependent inhibition of GAPDH activity resulted in an increase of MG levels in a human red blood cell culture (Beisswenger et al., 2003) and that the rapid inactivation of GAPDH is mediated by the interaction of MG with arginine residues in enzymes (Leoncini et al., 1981).

In addition to the increased carbohydrate levels and glycolysis in NH_4_^+^-grown plants, several TCA cycle enzymes were induced to counteract the depletion of TCA intermediates diverted to NH_4_^+^ assimilation; NADP^+^-IDH activity was enhanced, and citrate synthase (CS), succinyl-CoA ligase (SCoAL), fumarase (FUM) mRNA, and pyruvate dehydrogenase complex (PDC) levels were increased (Supplementary Figure S1). In previous studies, NH_4_^+^ nutrition led to increased mitochondrial PDC activity in the leaf tissue of sugar beet and in the root tissue of pea (Raab and Terry, 1995; Lasa et al., 2002b), as well as upregulation of *PDC1* in *Arabidopsis* shoots (Hachiya et al., 2012). Sarasketa et al. (2016) reported that TCA cycle and anaplerotic reaction enzymes, such as NADP^+^-IDH, malate dehydrogenase (MDH), NAD^+^-dependent malic enzyme (NAD-ME), and NADP^+^-dependent malic enzyme (NADP-ME), were also induced in NH_4_^+^-grown *Arabidopsis*.

### High MG Content Induces the Glyoxalase Pathway

Because MG-catabolizing enzymes are substrate-inducible (Kaur et al., 2016), high MG levels accelerate their own detoxification, indicating the presence of fine-tuned regulatory mechanisms responsible for the regulation of cellular MG concentration. In the present study, NH_4_^+^ nutrition resulted in higher GLXI and GLXII activity and induction of genes encoding some GLXI/II (**Figures 4, 5**). The higher activity of GLXI in leaf extracts and isolated chloroplasts in NH_4_^+^-grown plants compared with that in NO_3_^–^-grown plants (**Figures 4A, 5A**) was related to higher expression of *GLXI.3* (**Figure 4C**), whereas higher GLXII activity in leaf extracts from NH_4_^+^-grown plants compared with that in NO_3_^–^-grown plants (**Figure 4B**) was associated with induction of the *GLXII.5* gene (**Figure 4C**). Our results suggest that under ammonium nutrition, the GLXII.5 isoform is targeted mainly to the chloroplast, as higher *GLXII.5* expression was accompanied by higher chlGLXII activity but lower mtGLXII activity in NH_4_^+^-grown plants compared with that in NO_3_^–^-grown plants (**Figures 5B, 6A**).

Although the gene expression levels and activities were increased in the glyoxalase pathway of NH_4_^+^-grown plants, they were insufficient to maintain the MG concentration at the low levels observed in NO_3_^–^-grown plants. Therefore, MG is a potent glycation agent that causes detrimental modifications to the *Arabidopsis* proteome and could play an important role in signal transduction, probably as a stress signal molecule (Kaur et al., 2015a; Hoque et al., 2016), as well as in plant adaptation to NH_4_^+^. Previously, it was proposed that carbonylated peptides derived from proteolytic degradation of irreversibly oxidized proteins can be a specific signal of oxidative stress (Møller and Sweetlove, 2010).

### Reduced Efficiency of Mitochondrial Catabolism of SLG and D-Lactate

Cellular D-lactate content was unchanged in response to NH_4_^+^ nutrition (**Figure 6D**). In plants, externally supplied D-lactate enters mitochondria via a putative D-lactate/H^+^ symporter or a D-lactate/malate antiporter (Atlante et al., 2005; de Bari et al., 2005). In the present study, CINN, an inhibitor of lactate and pyruvate transport through cell membranes mediated by monocarboxylate transporters (MCTs) in animals (Ling et al., 2012), did not inhibit D-lactate uptake by *A. thaliana* mitochondria. These results were in agreement with those reported for mitochondria isolated from *Helianthus tuberosus* (Atlante et al., 2005), thus indicating that CINN is an improper inhibitor for plant D-lactate transporters. Alternatively, SLG, an upstream intermediate of the MG side pathway of glycolysis, may be transported into mitochondria, where D-lactate is produced by mtGLXII. The activity of D-LDH, which catalyzes the oxidation of D-lactate to pyruvate using cyt *c* as an electron acceptor, and that of mtGLXII were lower in NH_4_^+^-grown plants compared with those in NO_3_^–^-grown plants (**Figures 6A,B**). Moreover, mitochondrial oxidation of externally supplied D-lactate and SLG was lower in response to NH_4_^+^ nutrition (**Figure 6C**). Oxygen consumption by isolated mitochondria supplemented with D-lactate and SLG was at similar levels in NH_4_^+^-grown plants, indicating the existence of a bottleneck at the level of the reaction catalyzed by mtGLXII.

Our results indicate that D-LDH activity was not limited by the low availability of the oxidized form of cyt *c* because the activity of complex III (the main pathway delivering electrons to cyt *c*) was lower (**Figure 6F**). Additionally, cyt *c* protein levels were higher in NH_4_^+^-grown plants (**Figure 6E**) and complex IV capacity did not change in response to NH_4_^+^ nutrition (Podgórska et al., 2013). It has been demonstrated that both genes that encode *Arabidopsis* cyt *c* protein, *CYTC-1* and *CYTC-2*, are positively regulated by NH_4_^+^ at the transcriptional level and also upregulated by soluble carbohydrates such as Glc, Fru, and Suc (Welchen et al., 2002). Thus, the higher cyt *c* protein levels could be attributed to higher levels of soluble carbohydrates (**Figure 2**) in response to NH_4_^+^ nutrition. In our study, the increased MG level (**Figure 3F**) correlated with higher levels of cyt *c* protein (**Figure 6E**). Welchen et al. (2016) demonstrated that higher levels of cyt *c* allow plants to overcome the stress imposed by high levels of MG. However, the lower D-LDH activity (**Figure 6B**) points to another possible important role of cyt *c* under ammonium deficiency that needs to be further studied.

NH_4_^+^-grown plants showed lower complex III activity than NO_3_^–^-grown plants (**Figure 6F**). Wang et al. (2009) have shown that MG inhibits respiratory complex III activity in rat aortic smooth muscle cell mitochondria. Because alagebrium, an advanced glycation end products cross-link breaker, reversed all of the mitochondrial effects of MG, it was proposed that complex III is the major and selective target of MG in the mitochondria. Additionally, incubation of rat renal mitochondria with MG produced a concentration-dependent decrease in state 3 respiration and in respiratory complex III activity that were significantly correlated with the quantity of MG-H1 in mitochondrial proteins (Rabbani and Thornalley, 2008a). Among the seven proteins in the mitochondrial proteome that were suggested as susceptible targets of advanced glycation residue formation, two were components of complex III: core protein I and cytochrome *c_1_* of the cytochrome *bc_1_* complex. The electron-transport activity in complex III is inhibited by the produced *S*-carboxymethylation (Rabbani and Thornalley, 2008a). Additionally, mitochondria isolated from rats with chronic hyperglycemia exhibited a diminution of respiratory complex III activity that was significantly correlated with the quantity of MG-H1 in mitochondrial proteins (Rosca et al., 2005). Thus, the lower complex III activity in NH_4_^+^-grown plants probably resulted from MG toxicity through MAGEs formation.

### MAGEs in Proteins Are Elevated in NH_4_^+^-Grown Plants

**FIGURE 8:**
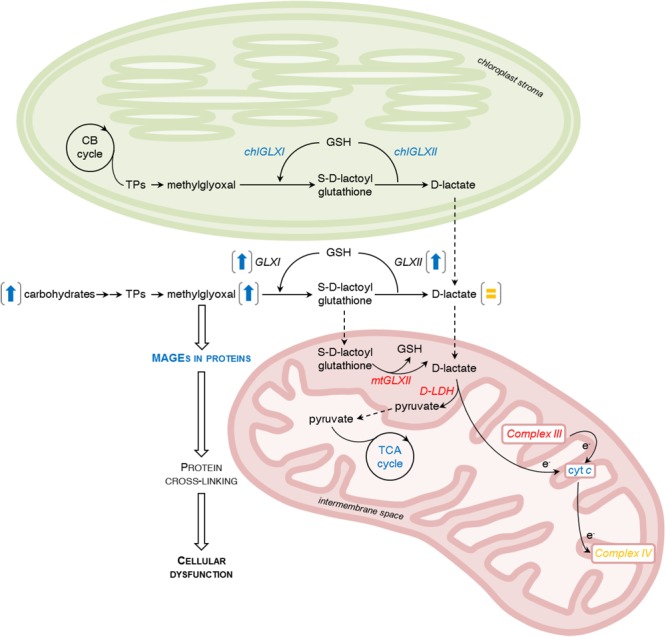
Influence of ammonium (NH_4_^+^) nutrition on methylglyoxal (MG) metabolism. MG production and accumulation are promoted by increased sugar concentration and enhanced glycolysis. MG may be produced also in chloroplasts from triose phosphate isomers (TPs) in the Calvin-Benson cycle (CB cycle). In tissue extracts, catabolism of MG in a glutathione (GSH)-dependent glyoxalase pathway comprised of glyoxalase I (GLXI) and glyoxalase II (GLXII) resulting in production of less toxic *S*-D-lactoylglutathione and D-lactate is enhanced. Additionally, chloroplastic isoforms of GLXI (chlGLXI) and GLXII (chlGLXII) have higher activity. In contrast, the cellular level of D-lactate remains stable. Mitochondrial metabolism of the MG-intermediates catabolic pathway is down-regulated. Mitochondrial glyoxalase II (mtGLXII) activity decreases. D-lactate is subsequently converted to pyruvate through the activity of mitochondrial D-lactate dehydrogenase (D-LDH) and electrons are transferred to the respiratory chain through cytochrome *c* (cyt *c*). In NH_4_^+^-grown plants, D-LDH activity is decreased, but not due to the low availability of the oxidized form of cytochrome *c* because cyt *c* protein levels in those plants are higher. Additionally, Complex III activity decreases and Complex IV activity remains stable. Pyruvate produced by D-LDH enters into the tricarboxylic acid (TCA) cycle that is enhanced to meet the demand of carbon skeletons for NH_4_^+^ assimilation. Over-accumulation of MG in the cell leads to formation of MG-derived advanced glycation end products (MAGEs). Modified proteins usually undergo structural and functional impairment that causes cellular dysfunction and mediates NH_4_^+^ toxicity. The difference in enzyme activities or metabolite levels between NH_4_^+^-grown and NO_3_^–^-grown plants are indicated by colors; increases are marked with blue, decreases with red, and lack of change with yellow letters. Results for whole leaf tissue extracts are presented in brackets as arrows (increases are marked with blue) or the equal sign (lack of change is marked with yellow).

The overproduction of MG led to MAGEs accumulation in proteins (**Figures 7A,B**), and the levels of MG-H1 modifications were higher, mainly in low-molecular-mass proteins (**Figure 7A**). In addition, the total CEL levels were higher in the leaf protein extracts of NH_4_^+^-grown plants compared with those of NO_3_^–^-grown plants (**Figure 7B**). Despite the fact that MG is derived from glycolysis, MG-H1 modifications of proteins are not positively correlated with carbohydrate concentrations (Supplementary Figure S4). The proposed model of MG-mediated toxicity in response to NH_4_^+^ nutrition is presented in **Figure 8**. Quantitative MAGEs analysis revealed that the *A. thaliana* proteome was dominated by arginine-derived modifications and that MG-H1 was the major adduct (Bilova et al., 2016). Bechtold et al. (2009) studied the *Arabidopsis* glycated proteome and showed that among all of the modifications, the MG-H1 content was the highest (approximately 2 mmol per mole of arginine residues); the content of CEL was 10-fold lower. In the *Arabidopsis* proteome, 502 proteins are constitutively glycated, and up to approximately 40% of the modifications are derived from MG such as MG-H1 and CEL (Bilova et al., 2016). Additionally, environmental cues (osmotic stress and excess light) may lead to an increase in the levels of specific advanced glycation end products (Bechtold et al., 2009; Paudel et al., 2016). In the present study, the major MG-H1 modification target in leaf tissue extracts was a protein with a molecular mass of approximately 50 kDa (**Figure 7A**), most probably the large subunit of Rubisco (∼56 kDa; Ma et al., 2009). Rubisco accounts for at least 20% of the total leaf protein weight (Cellar et al., 2008) and is the most abundant protein modified by advanced glycation end products in the *A. thaliana* proteome as shown by liquid chromatography-based bottom-up proteomic analysis (Bilova et al., 2016).

High MG accumulation exerts irreversible effects on protein structure associated with misfolding and cross-linking that usually lead to functional impairment (Silva et al., 2013; Paudel et al., 2016) because the functionally important arginine residues in proteins are often subjected to dicarbonyl glycation. Dicarbonyl stress reflects an irreversible damage to the proteome, which is associated with metabolic disorders (Rabbani and Thornalley, 2008b). Permanent modifications in the proteome require replenishment by unmodified proteins. One of the reported adverse effects of MG is the increased degradation of proteins (Rabbani and Thornalley, 2012; Li, 2016). In the present study, the higher activity of proteolytic enzymes in response to NH_4_^+^ nutrition (**Figure 7C**) might indicate the enhanced degradation of MAGE-modified proteins.

## Conclusion

Overall, our study indicates that NH_4_^+^ nutrition leads to MG accumulation and shows that MAGEs can be mediators of MG-induced damage.

## Author Contributions

MO-B designed and conducted MAGEs formation and proteolysis experiments. MO-B and KB conducted equal parts of the main experiments. BS with the help of M-NV and M-PH-S performed the TCA cycle and glycolytic enzyme studies. MO-B and KB analyzed the data. MO-B wrote the manuscript with the contribution of KB. BS conceived the project, designed most of the experiments, supervised all experiments, and complemented the writing.

## Conflict of Interest Statement

The authors declare that the research was conducted in the absence of any commercial or financial relationships that could be construed as a potential conflict of interest.

## Footnotes

1 https://www.arabidopsis.org/
