# Supplementary material for: Enhanced Formation of Methylglyoxal-Derived Advanced Glycation End Products in Arabidopsis Under Ammonium Nutrition
Source: Front Plant Sci. 2018 May 24;9:667. doi: 10.3389/fpls.2018.00667 (PMC5976750; doi:10.3389/fpls.2018.00667)
Supplement: Supplementary file 1 [file Data_Sheet_1.docx]

Supplementary Material

Enhanced formation of methylglyoxal-derived advanced glycation end products in *Arabidopsis* under ammonium nutrition

Klaudia Borysiuk^1§^, Monika Ostaszewska-Bugajska^1§^*, Marie-Noëlle Vaultier^2^, Marie-Paule Hasenfratz-Sauder^2^, Bożena Szal^1^*

^1^ Institute of Experimental Plant Biology and Biotechnology, Faculty of Biology, University of Warsaw, Miecznikowa 1, 02-096 Warsaw, Poland

^2^ Ecologie et Ecophysiologie Forestières, Université de Lorraine, INRA, UMR 1137, Vandoeuvre-lès-Nancy, F-54500, France

^§^ These authors contributed equally to the article.

***Correspondence**:

Corresponding authors

Bożena Szal

[szal@biol.uw.edu.pl](mailto:szal@biol.uw.edu.pl)

Tel: 0048225543005

Fax: 0048225542022

Monika Ostaszewska-Bugajska

[m.ostaszewska@biol.uw.edu.pl](mailto:m.ostaszewska@biol.uw.edu.pl)

Tel: 0048225543009

Fax: 0048225542022

# Supplementary Figures and Tables

## Supplementary Figures

*

*

*

*


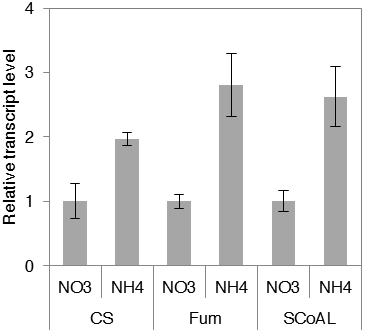


**A**

**B**

**C**


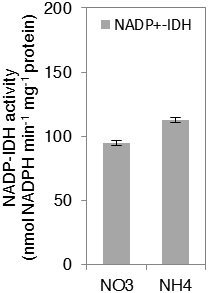


1.00 3.25*


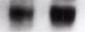


NO_3_ NH_4_

PDC

NO_3_ NH_4_

NO_3_ NH_4_ NO_3_ NH_4_ NO_3_ NH_4_

**Figure S1**. Influence of ammonium (NH_4_^+^) nutrition on mitochondrial matrix-localized enzymes. (A) Activity of NADP^+^-dependent isocitrate dehydrogenase (NADP^+^-IDH). (B) Transcript levels of citrate synthase (CS), fumarase (Fum), and succinyl-CoA ligase (SCoAL). (C) Protein levels of pyruvate dehydrogenase complex (PDC) in nitrate (NO_3_^-^)-grown and NH_4_^+^-grown plants. Mitochondrial protein (10 μg) was loaded, separated by sodium dodecyl sulfate-polyacrylamide gel electrophoresis, immunoblotted, and visualized by chemiluminescence. Signal intensity corresponding to PDC (molecular mass, approximately 43 kDa) versus VDAC1, which was used for protein level normalization, was estimated using Quantity One 4.6.2 after background correction. Results are expressed relative to the control; the amount of protein in mitochondria isolated from leaf tissue of NO_3_^-^-grown plants was set at 1.00. Representative results are shown. Values are the mean ± standard deviation (SD) of 3 or 4 biological and 1-2 technical replicates. Significant differences (*P* ≤ 0.05) between NO_3_^-^-grown and NH_4_^+^-grown plants are indicated by an asterisk (*).


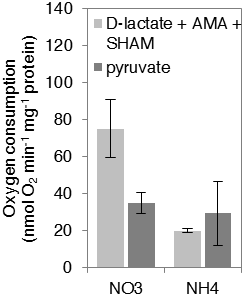


*

NO_3_ NH_4_

**Figure S2.** Mitochondrial oxygen consumption control measurements. Isolated and purified mitochondria from leaf tissue of in nitrate (NO_3_^-^)-grown and ammonium (NH_4_^+^)-grown plants were used. Values are the mean ± standard deviation (SD) of 2 biological replicates. Significant differences (*P* ≤ 0.05) between NO_3_^-^-grown and NH_4_^+^-grown plants are indicated by an asterisk (*). AMA, antimycin A; SHAM, salicylhydroxamic acid.


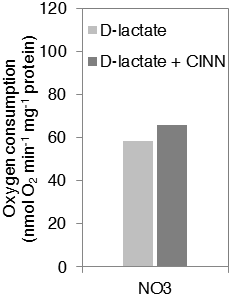


NO_3_

**Figure S3.** Mitochondrial oxygen consumption measurements with α-cyano-4-hydroxycinnamate (CINN) and D-lactate as a respiratory substrate. Isolated and purified mitochondria from leaf tissue of nitrate (NO_3_^-^)-grown plants were used. Representative results from one experiment are shown. Mitochondria were preincubated with CINN (5 mM), then substrate (5 mM) and ADP (80 µM) were added. Respiratory rates in state 3 are presented.


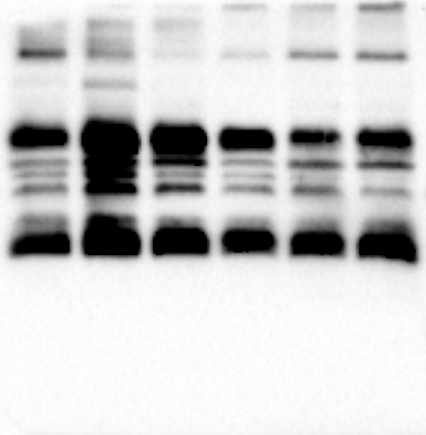

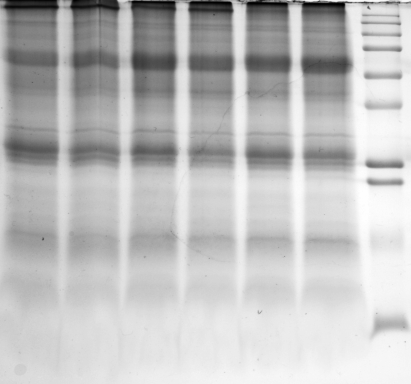


Total protein

WT *fro1 +*N –N +S -S

WT *fro1 +*N –N +S -S

MG-H1

**A**

**B**

ND


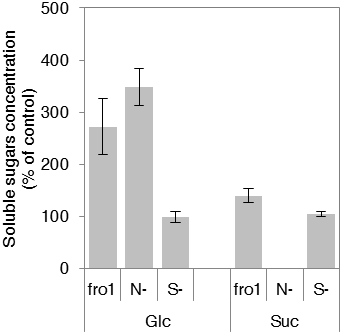


**Figure S4.** Relation between different levels of carbohydrates and methylglyoxal (MG)-derived advanced glycation end products formation in proteins. (A) Concentration of glucose (Glc) and sucrose (Suc), (B) MG-derived hydroimidazolone 1 (MG-H1) levels in proteins and total protein stained gel. *A. thaliana* *frostbite 1* (respiratory chain Complex I lacking mutant, *fro1*) plants and control plants ecotype C24 (WT) grown in non-stress conditions; *A. thaliana* ecotype Columbia-0 stressed by nitrate or sulphur deficiency [1 mM nitrate (-N) or 10 mM nitrate (+N) and plants grown on sulphur-depleted Knopp medium (-S) or in presence of 1.25 mM SO_4_^2-^ (+S). ND, not determined. Values are the mean ± standard deviation (SD) of 3 biological and 2 technical replicates.

| Gene | AGI code | Forward Primer | Reverse Primer |
| --- | --- | --- | --- |
| CS | At2g44350 | 5’– AGCAGGACCGTCTGAAGAAA – 3’ | 5’– CACAAAAGACCCTCCGGTAA – 3’ |
| SCoAL | At5g08300 | 5’– AAAAGCTCGCCGTTGTTCTA – 3’ | 5’– GTCACTCCTGCCACCATTTT – 3’ |
| Fum | At2g47510 | 5’– CTCACACTCAAGATGCTACACC – 3’ | 5’– TGCAGTGACAAATGGCAAGT – 3’ |
| GLXI.1 | At1g67280 | 5’– CTGTCAAAGGTGGCAAAACTGT – 3’ | 5’– GGCTATTGTGTACTTGTACTCTGG – 3’ |
| GLXI.2 | At1g11840 | 5’– CGGCACCAAGATTGTCTCATT – 3’ | 5’– ACAAAAGAAACGAGCACACACA – 3’ |
| GLXI.3 | At1g08110 | 5’– GTCATTGCTGAAGAGGCTGGA – 3’ | 5’– GAGGCTCAGAGTTCCCATTG – 3’ |
| GLXII.2 | At3g10850 | 5’– GTTGATAAGCCAGAGATACAGGAGAA – 3’ | 5’– TACACAAAACATAGGCAAGAAGC – 3’ |
| GLXII.4 | At1g06130 | 5’– TCATTCTTTAGATGTTCTAGGAAGC – 3’ | 5’– TACCAGCATCACGCAAAGTC – 3’ |
| GLXII.5 | At2g31350 | 5’– TGAACTGGTGCCTTGCCTTA – 3’ | 5’– AGCTTCAGAAGGGTCAACCA – 3’ |

## Supplementary Tables

**Table S1**. List of qRT-PCR primers used in this study.

In the table glyoxalase genes nomenclature according to Schmitz et al. (2017) is used. In paper by Mustafiz et al. (2011) GLXI.1 was named GLYI.6; GLXI.2 was named GLYI.3; GLXI.3 was named GLYI.2; GLXII.2 was named GLYII.5; GLXII.4 was named GLYII.1 and GLXII.5 was named GLYII.3.

**Table S2**. Densitometric analysis of hydroimidazolone 1 (MG-H1) levels in proteins on Figure 7A. Mean signal intensities of the bands from three replicates are expressed relative to the value for the control (NO_3_^-^-grown plants), which was normalized to unity (1.00). *P* values were obtained using two-tailed Student’s t-test. NO3, NO_3_^-^-grown plants; NH4, NH_4_^+^-grown plants.

| Band No. | NO_3_ | NH_4_ | *P value* |
| --- | --- | --- | --- |
| 1 | 1.00 ± 0.03 | 1.16 ± 0.07 | *P* ≤ 0.1 |
| 2 | 1.00 ± 0.05 | 0.75 ± 0.02 | *P* ≤ 0.05 |
| 3 | 1.00 ± 0.14 | 1.29 ± 0.10 | *P* ≤ 0.1 |
| 4 | 1.00 ± 0.08 | 1.59 ± 0.14 | *P* ≤ 0.05 |
| 5 | 1.00 ± 0.31 | 1.71 ± 0.23 | *P* ≤ 0.05 |
| 6 | 1.00 ± 0.27 | 0.63 ± 0.09 | *P* ≤ 0.05 |
| 7 | 1.00 ± 0.24 | 1.62 ± 0.24 | *P* ≤ 0.05 |
